# Supplementary material for: Clinical Utility of Oncuria™, a Multiplexed Liquid Biopsy for the Non-Invasive Detection of Bladder Cancer—A Pilot Study
Source: Diagnostics (Basel). 2022 Jan 6;12(1):131. doi: 10.3390/diagnostics12010131 (PMC8774623; doi:10.3390/diagnostics12010131)
Supplement: Supplementary file 1 [file diagnostics-12-00131-s001.zip › Supplementary File S1.pdf]

Urine-based biomarkers, Clinical Utility Study

UROLOGIST DEMOGRAPHICS QUESTIONNAIRE

Please choose the response that best describes you (Circle all that apply).

1. Please review the informed consent associated with this study attached in the email from us. By completing this survey, you are providing consent to participate in this study entitled 'Clinical utility of a non-invasive urine test for evaluating bladder cancer patients'. Please enter today's date and time to continue.

Date / Time

Date MM/DD/YYYY Time hh mm AM/PM -

2. Ethnicity

- ☐ Hispanic or Latino
- ☐ Non-Hispanic or Latino

3. Race

- ☐ African American
- ☐ Asian
- ☐ Caucasian
- ☐ Native American
- ☐ Native Hawaiian/Pacific Islander
- ☐ Other

4. Gender

- ☐ Male
- ☐ Female

5. Age

- ☐ < 30 years
- ☐ 30-45 years
- ☐ 46-60 years
- ☐ > 60 years

6. Degree

- [ ] MD
- [ ] DO
- [ ] Other (please specify)

- [ ] None of the above

7. Practice speciality

- [ ] General Urology
- [ ] Urologic Oncology
- [ ] Urolithiasis/Endourology
- [ ] Pediatric Urology
- [ ] Pelvic Medicine and Reconstruction
- [ ] Male Impotence and Infertility
- [ ] Minimal Invasive/Robotic Surgery
- [ ] Other (please specify)

8. Practice Type

- [ ] Solo
- [ ] Group/private practice
- [ ] Group/university practice
- [ ] Other (please specify)

- [ ] None of the above

9. Office setting

- [ ] Rural
- [ ] Urban
- [ ] Other (please specify)

10. Percentage of Medicare patients in your practice

- [ ] < 25%
- [ ] 26-50%
- [ ] > 50%
- [ ] Other (please specify)

- [ ] None of the above

11. Percentage of Medicaid patients in your practice

- [ ] < 25%
- [ ] 26-50%
- [ ] > 50%
- [ ] Other (please specify)

- [ ] None of the above

12. Up to 15 practicing urologists who complete this demographics survey will be invited to participate in a one-on-one interview on Zoom in which 9 clinical vignettes related to urinary biomarkers for bladder cancer detection will be reviewed. If you are interested in further participation and to qualify for the $250 gift certificate, then please provide your name and best contact information below. We will contact you shortly to schedule the Zoom meeting.

Full name:

Email address:

Office Telephone:

Clinical utility of a non-invasive urine test for evaluating bladder cancer patients

Oncuria™

To familiarize yourself with Oncuria™, please read the following:

- Analytical validation study and
- Clinical validation study

Gross Hematuria

- Mrs. GA is a 66 year-old non-white female who presented with painless gross hematuria. Seen by PCP, thought to have a UTI. Treated with antibiotics with improvement, then recurrence of gross hematuria 2 months later. UC - negative. Denies trauma. No pain or symptoms.
- PMH - borderline DM
- MEDS - None
- SHx- Former smoker (46 pack years)
- PE
  - Negative
- Urinalysis
  - Reddish color
  - Dipstick: - nitrite, - leukocyte esterase and moderate blood
  - Microscopic - TNTC RBC/hpf, 2 WBC/hpf, - bacteria

- Mrs. GA is a 66 year-old non-white female who presented with painless gross hematuria. Seen by PCP, thought to have a UTI. Treated with antibiotics with improvement, then recurrence of gross hematuria 2 months later. UC - negative. Denies trauma. No pain or symptoms
- PMH - borderline DM
- MEDS - None
- SHx- Former smoker (46 pack years)
- PE
  - Negative
- Urinalysis
  - Reddish color
  - Dipstick: - nitrite, - leukocyte esterase and moderate blood
  - Microscopic - TNTC RBC/hpf, 2 WBC/hpf, - bacteria

- **What urine-based test would you order next and why?**

❑Urinary Cytology

❑Immunocyt/uCyt+

❑Urovysion (FISH)

❑BTAstat

❑Bladdercheck (NMP22)

❑Other; ___________________

❑None

- Mrs. GA is a 66 year-old non-white female who presented with painless gross hematuria. Seen by PCP, thought to have a UTI. Treated with antibiotics with improvement, then recurrence of gross hematuria 2 months later. UC - negative. Denies trauma. No pain or symptoms
- PMH - borderline DM
- MEDS - None
- SHx- Former smoker (46 pack years)
- PE
  - Negative
- Urinalysis
  - Reddish color
  - Dipstick: - nitrite, - leukocyte esterase and moderate blood
  - Microscopic - TNTC RBC/hpf, 2 WBC/hpf, - bacteria
- Voided urinary cytology - negative

- **What additional urine-based test would you order next and why?**

❑Immunocyt/uCyt+

❑Urovysion (FISH)

❑BTAstat

❑Bladdercheck (NMP22)

❑Other; ____________________

❑None

- Mrs. GA is a 66 year-old non-white female who presented with painless gross hematuria. Seen by PCP, thought to have a UTI. Treated with antibiotics with improvement, then recurrence of gross hematuria 2 months later. UC - negative. Denies trauma. No pain or symptoms
- PMH - borderline DM
- MEDS - None
- SHx- Former smoker (46 pack years)
- PE
  - Negative
- Urinalysis
  - Reddish color
  - Dipstick: - nitrite, - leukocyte esterase and moderate blood
  - Microscopic - TNTC RBC/hpf, 2 WBC/hpf, - bacteria
- Voided urinary cytology - negative

- **What diagnostic test(s) would you perform next?**

❑ Office cystoscopy

❑ CT scan of abdomen/pelvis

❑ Transurethral resection of bladder tumor (TURBT)

❑ Other; ____________________

❑ None

- Mrs. GA is a 66 year-old non-white female who presented with painless gross hematuria. Seen by PCP, thought to have a UTI. Treated with antibiotics with improvement, then recurrence of gross hematuria 2 months later. UC - negative. Denies trauma. No pain or symptoms
- PMH - borderline DM
- MEDS - None
- SHx- Former smoker (46 pack years)
- PE
  - Negative
- Urinalysis
  - Reddish color
  - Dipstick: - nitrite, - leukocyte esterase and moderate blood
  - Microscopic - TNTC RBC/hpf, 2 WBC/hpf, - bacteria
- Oncuria risk score 84% (positive)

Now, noting the Oncuria risk score, would this change the urine-based test you ordered?

❑Yes ❑No

If yes, then what would you order and why?

❑Urinary Cytology

❑Immunocyt/uCyt+

❑Urovysion (FISH)

❑BTAstat

❑Bladdercheck (NMP22)

❑Other; ______________________

❑None

- Mrs. GA is a 66 year-old non-white female who presented with painless gross hematuria. Seen by PCP, thought to have a UTI. Treated with antibiotics with improvement, then recurrence of gross hematuria 2 months later. UC - negative. Denies trauma. No pain or symptoms
- PMH - borderline DM
- MEDS - None
- SHx- Former smoker (46 pack years)
- PE
  - Negative
- Urinalysis
  - Reddish color
  - Dipstick: - nitrite, - leukocyte esterase and moderate blood
  - Microscopic - TNTC RBC/hpf, 2 WBC/hpf, - bacteria
- Oncuria risk score 84% (positive)

- **Now, noting the Oncuria risk score, what diagnostic tests would you perform next?**

❑ Office cystoscopy

❑ CT scan of abdomen/pelvis

❑ Transurethral resection of bladder tumor (TURBT)

❑ Other;________________

❑ None

FINAL RESULT:
Cytology is negative; bladderchek (NMP22) is positive
Office cystoscopy noted bladder mass
CT scan noted bladder mass and upper tracts (kidneys) were normal.
TURBT noted a 5 cm tumor - pathology Ta HG TCC (UC)

If clinically launched and is as sensitive as described in the manuscripts, how could Oncuria change the way you use urine-based bladder cancer detection assays?
Did or could Oncuria change the way you practice based on this case?

- Mr. MA is a 56 year-old white male who presented with intermittent painless gross hematuria over the past weekend. Denies trauma. No other symptoms.
- PMH - BPH
- MEDS - Flomax
- SHx- Has smoked cigars and from a household that smoked, works in customer service
- PE
  - Negative
- Urinalysis
  - Dark amber color
  - Dipstick: - nitrite, - leukocyte esterase and trace blood
  - Microscopic - 50-100 RBC/hpf, 1-2 WBC/hpf, - bacteria

- Mr. MA is a 56 year-old white male who presented with intermittent painless gross hematuria over the past weekend. Denies trauma. No other symptoms
- PMH - BPH
- MEDS - Flomax
- SHx- Has smoked cigars and from a household that smoked, works in customer service
- PE
  - Negative
- Urinalysis
  - Dark amber color
  - Dipstick: - nitrite, - leukocyte esterase and trace blood
  - Microscopic - 50-100 RBC/hpf, 1-2 WBC/hpf, - bacteria

- **What urine-based test would you order next and why?**

- ❑ Urinary Cytology
- ❑ Immunocyt/uCyt+
- ❑ Urovysion (FISH)
- ❑ BTAstat
- ❑ Bladdercheck (NMP22)
- ❑ Other; ________________
- ❑ None

- Mr. MA is a 56 year-old white male who presented with intermittent painless gross hematuria over the past weekend. Denies trauma. No other symptoms
- PMH - BPH
- MEDS - Flomax
- SHx- Has smoked cigars and from a household that smoked, works in customer service
- PE
  - Negative
- Urinalysis
  - Dark amber color
  - Dipstick: - nitrite, - leukocyte esterase and trace blood
  - Microscopic - 50-100 RBC/hpf, 1-2 WBC/hpf, - bacteria
- Voided urinary cytology - negative

- **What additional urine-based test would you order next and why?**

- ❑ Immunocyt/uCyt+
- ❑ Urovysion (FISH)
- ❑ BTAstat
- ❑ Bladdercheck (NMP22)
- ❑ Other; ________________
- ❑ None

- Mr. MA is a 56 year-old white male who presented with intermittent painless gross hematuria over the past weekend. Denies trauma. No other symptoms
- PMH - BPH
- MEDS - Flomax
- SHx- Has smoked cigars and from a household that smoked, works in customer service
- PE
  - Negative
- Urinalysis
  - Dark amber color
  - Dipstick: - nitrite, - leukocyte esterase and trace blood
  - Microscopic - 50-100 RBC/hpf, 1-2 WBC/hpf, - bacteria
- Voided urinary cytology - negative

- **What diagnostic test(s) would you perform next?**

❑ Office cystoscopy

❑ CT scan of abdomen/pelvis

❑ Transurethral resection of bladder tumor (TURBT)

❑ Other; ________________

❑ None

- Mr. MA is a 56 year-old white male who presented with intermittent painless gross hematuria over the past weekend. Denies trauma. No other symptoms
- PMH - BPH
- MEDS - Flomax
- SHx- Has smoked cigars and from a household that smoked, works in customer service
- PE
  - Negative
- Urinalysis
  - Dark amber color
  - Dipstick: - nitrite, - leukocyte esterase and trace blood
  - Microscopic - 50-100 RBC/hpf, 1-2 WBC/hpf, - bacteria
- Oncuria risk score 18% (negative)

Now, noting the Oncuria risk score, would this change the urine-based test you ordered?

❑Yes ❑No

If yes, then what would you order and why?

❑Urinary Cytology

❑Immunocyt/uCyt+

❑Urovysion (FISH)

❑BTAstat

❑Bladdercheck (NMP22)

❑Other; ______________________

❑None

- Mr. MA is a 56 year-old white male who presented with intermittent painless gross hematuria over the past weekend. Denies trauma. No other symptoms
- PMH - BPH
- MEDS - Flomax
- SHx- Has smoked cigars and from a household that smoked, works in customer service
- PE
  - Negative
- Urinalysis
  - Dark amber color
  - Dipstick: - nitrite, - leukocyte esterase and trace blood
  - Microscopic - 50-100 RBC/hpf, 1-2 WBC/hpf, - bacteria
- Oncuria risk score 18% (negative)

- **Now, noting the Oncuria risk score, what diagnostic tests would you perform next?**

❑ Office cystoscopy

❑ CT scan of abdomen/pelvis

❑ Transurethral resection of bladder tumor (TURBT)

❑ Other;________________

❑ None

FINAL RESULT:
Cytology and bladdercheck (NMP22) negative
Office cystoscopy was negative
CT scan abdomen and pelvis was negative.

If clinically launched and is as sensitive as described in the manuscripts, how could Oncuria change the way you use urine-based bladder cancer detection assays?
Did or could Oncuria change the way you practice based on this case?

- Mr. JT is a 52 year-old homeless white male who presented to the ED with painless gross hematuria for 24 hours. Lost to follow-up for 2 months, then presented back to the ED with painless gross hematuria. Denies trauma. No pain or symptoms.
- PMH - None
- MEDS - None
- SHx- Current smoker (74 pack years) and methamphetamine abuser
- PE
  - Negative
- Urinalysis
  - Red with blood clots noted
  - Dipstick: - nitrite, - leukocyte esterase and large blood
  - Microscopic - TNTC RBC/hpf, - WBC/hpf, - bacteria

- Mr. JT is a 52 year-old homeless white male who presented to the ED with painless gross hematuria for 24 hours. Lost to follow-up for 2 months, then presented back to the ED with painless gross hematuria. Denies trauma. No pain or symptoms
- PMH - None
- MEDS - None
- SHx- Current smoker (74 pack years) and methamphetamine abuser
- PE
  - Negative
- Urinalysis
  - Red with blood clots noted
  - Dipstick: - nitrite, - leukocyte esterase and large blood
  - Microscopic - TNTC RBC/hpf, - WBC/hpf, - bacteria

- **What urine-based test would you order next and why?**

❑ Urinary Cytology

❑ Immunocyt/uCyt+

❑ Urovysion (FISH)

❑ BTAstat

❑ Bladdercheck (NMP22)

❑ Other; ____________________

❑ None

- Mr. JT is a 52 year-old homeless white male who presented to the ED with painless gross hematuria for 24 hours. Lost to follow-up for 2 months, then presented back to the ED with painless gross hematuria. Denies trauma. No pain or symptoms
- PMH - None
- MEDS - None
- SHx- Current smoker (74 pack years) and methamphetamine abuser
- PE
  - Negative
- Urinalysis
  - Red with blood clots noted
  - Dipstick: - nitrite, - leukocyte esterase and large blood
  - Microscopic - TNTC RBC/hpf, - WBC/hpf, - bacteria
- Voided urinary cytology - atypia

- **What additional urine-based test would you order next and why?**

❑ Immunocyt/uCyt+

❑ Urovysion (FISH)

❑ BTAstat

❑ Bladdercheck (NMP22)

❑ Other; ____________________

❑ None

- Mr. JT is a 52 year-old homeless white male who presented to the ED with painless gross hematuria for 24 hours. Lost to follow-up for 2 months, then presented back to the ED with painless gross hematuria. Denies trauma. No pain or symptoms
- PMH - None
- MEDS - None
- SHx- Current smoker (74 pack years) and methamphetamine abuser
- PE
  - Negative
- Urinalysis
  - Red with blood clots noted
  - Dipstick: - nitrite, - leukocyte esterase and large blood
  - Microscopic - TNTC RBC/hpf, - WBC/hpf, - bacteria
- Voided urinary cytology - atypia

- **What diagnostic test(s) would you perform next?**

- ❑ Office cystoscopy
- ❑ CT scan of abdomen/pelvis
- ❑ Transurethral resection of bladder tumor (TURBT)
- ❑ Other; ____________________
- ❑ None

- Mr. JT is a 52 year-old homeless white male who presented to the ED with painless gross hematuria for 24 hours. Lost to follow-up for 2 months, then presented back to the ED with painless gross hematuria. Denies trauma. No pain or symptoms
- PMH - None
- MEDS - None
- SHx- Current smoker (74 pack years) and methamphetamine abuser
- PE
  - Negative
- Urinalysis
  - Red with blood clots noted
  - Dipstick: - nitrite, - leukocyte esterase and large blood
  - Microscopic - TNTC RBC/hpf, - WBC/hpf, - bacteria
- Oncuria risk score 63% (positive)

Now, noting the Oncuria risk score, would this change the urine-based test you ordered?

❑Yes ❑No

If yes, then what would you order and why?

❑Urinary Cytology

❑Immunocyt/uCyt+

❑Urovysion (FISH)

❑BTAstat

❑Bladdercheck (NMP22)

❑Other; ______________________

❑None

- Mr. JT is a 52 year-old homeless white male who presented to the ED with painless gross hematuria for 24 hours. Lost to follow-up for 2 months, then presented back to the ED with painless gross hematuria. Denies trauma. No pain or symptoms
- PMH - None
- MEDS - None
- SHx- Current smoker (74 pack years) and methamphetamine abuser
- PE
  - Negative
- Urinalysis
  - Red with blood clots noted
  - Dipstick: - nitrite, - leukocyte esterase and large blood
  - Microscopic - TNTC RBC/hpf, - WBC/hpf, - bacteria
- Oncuria risk score 63% (positive)

- **Now, noting the Oncuria risk score, what diagnostic tests would you perform next?**

❑Office cystoscopy

❑CT scan of abdomen/pelvis

❑Transurethral resection of bladder tumor (TURBT)

❑Other;____________________

❑None

FINAL RESULT:
Cytology atypia and bladderchek (NMP22) positive
Office cystoscopy noted a bladder mass
CT scan noted bladder mass and negative upper tract
TURBT demonstrated a 7 cm mass - pathology T1 HG TCC (UC)

If clinically launched and is as sensitive as described in the manuscripts, how could Oncuria change the way you use urine-based bladder cancer detection assays?
Did or could Oncuria change the way you practice based on this case?

Microscopic Hematuria

- Mrs. MH is a 63 year-old white female found to have microscopic hematuria on annual employment physical. Persistence of microscopic hematuria for 6 months. No pain or symptoms.
- PMH - Type II DM, HTN
- MEDS - Metformin, Lisinopril
- SHx- Never smoker, works in histology laboratory with chemicals
- PE
  - Negative
- Urinalysis
  - Straw color
  - Dipstick: - nitrite, - leukocyte esterase and moderate blood
  - Microscopic - 5 RBC/hpf (6 months earlier 3 RBC/hpf), 0 WBC/hpf or bacteria
- Urine culture
  - 10,000 normal flora

- Mrs. MH is a 63 year-old white female found to have microscopic hematuria on annual employment physical. Persistence of microscopic hematuria for 6 months. No pain or symptoms.
- PMH - Type II DM, HTN
- MEDS - Metformin, Lisinopril
- SHx- Never smoker, works in histology laboratory with chemicals
- PE
  - Negative
- Urinalysis
  - Straw color
  - Dipstick: - nitrite, - leukocyte esterase and moderate blood
  - Microscopic - 5 RBC/hpf (6 months earlier 3 RBC/hpf), 0 WBC/hpf or bacteria
- Urine culture
  - 10,000 normal flora

- **What urine-based test would you order next and why?**

❑ Urinary Cytology

❑ Immunocyt/uCyt+

❑ Urovysion (FISH)

❑ BTAstat

❑ Bladdercheck (NMP22)

❑ Other; ____________________

❑ None

- Mrs. MH is a 63 year-old white female found to have microscopic hematuria on annual employment physical. Persistence of microscopic hematuria for 6 months. No pain or symptoms.
- PMH - Type II DM, HTN
- MEDS - Metformin, Lisinopril
- SHx- Never smoker, works in histology laboratory with chemicals
- PE
  - Negative
- Urinalysis
  - Straw color
  - Dipstick: - nitrite, - leukocyte esterase and moderate blood
  - Microscopic - 5 RBC/hpf (6 months earlier 3 RBC/hpf), 0 WBC/hpf or bacteria
- Urine culture
  - 10,000 normal flora
- Voided urinary cytology - negative

- **What additional urine-based test would you order next and why?**

❑Immunocyt/uCyt+

❑Urovysion (FISH)

❑BTAstat

❑Bladdercheck (NMP22)

❑Other; ____________________

❑None

- Mrs. MH is a 63 year-old white female found to have microscopic hematuria on annual employment physical. Persistence of microscopic hematuria for 6 months. No pain or symptoms.
- PMH - Type II DM, HTN
- MEDS - Metformin, Lisinopril
- SHx- Never smoker, works in histology laboratory with chemicals
- PE
  - Negative
- Urinalysis
  - Straw color
  - Dipstick: - nitrite, - leukocyte esterase and moderate blood
  - Microscopic - 5 RBC/hpf (6 months earlier 3 RBC/hpf), 0 WBC/hpf or bacteria
- Urine culture
  - 10,000 normal flora
- Voided urinary cytology - negative

- **What diagnostic test(s) would you perform next?**

❑Office cystoscopy

❑CT scan of abdomen/pelvis

❑Transurethral resection of bladder tumor (TURBT)

❑Other; ____________________

❑None

- Mrs. MH is a 63 year-old white female found to have microscopic hematuria on annual employment physical. Persistence of microscopic hematuria for 6 months. No pain or symptoms.
- PMH - Type II DM, HTN
- MEDS - Metformin, Lisinopril
- SHx- Never smoker, works in histology laboratory with chemicals
- PE
  - Negative
- Urinalysis
  - Straw color
  - Dipstick: - nitrite, - leukocyte esterase and moderate blood
  - Microscopic - 5 RBC/hpf (6 months earlier 3 RBC/hpf), 0 WBC/hpf or bacteria
- Urine culture
  - 10,000 normal flora
- Oncuria risk score 7% (negative)

Now, noting the Oncuria risk score, would this change the urine-based test you ordered?

❑Yes ❑No

If yes, then what would you order and why?

❑Urinary Cytology

❑Immunocyt/uCyt+

❑Urovysion (FISH)

❑BTAstat

❑Bladdercheck (NMP22)

❑Other; ____________________

❑None

- Mrs. MH is a 63 year-old white female found to have microscopic hematuria on annual employment physical. Persistence of microscopic hematuria for 6 months. No pain or symptoms.
- PMH - Type II DM, HTN
- MEDS - Metformin, Lisinopril
- SHx- Never smoker, works in histology laboratory with chemicals
- PE
  - Negative
- Urinalysis
  - Straw color
  - Dipstick: - nitrite, - leukocyte esterase and moderate blood
  - Microscopic - 5 RBC/hpf (6 months earlier 3 RBC/hpf), 0 WBC/hpf or bacteria
- Urine culture
  - 10,000 normal flora
- Oncuria risk score 7% (negative)

- **Now, noting the Oncuria risk score, what diagnostic tests would you perform next?**

- [ ] Office cystoscopy
- [ ] CT scan of abdomen/pelvis
- [ ] Transurethral resection of bladder tumor (TURBT)
- [ ] Other;____________________
- [ ] None

FINAL RESULT:
Cytology and bladderchek (NMP22) negative
Office cystoscopy negative
CT scan negative

If clinically launched and is as sensitive as described in the manuscripts, how could Oncuria change the way you use urine-based bladder cancer detection assays?
Did or could Oncuria change the way you practice based on this case?

- Mr. TW is a 71 year-old white male found to have microscopic hematuria on 2 urinalyses after recent hospitalization for exacerbation of congestive heart failure (CHF). No pain or symptoms noted.
- PMH - CHF, HTN, panic attacks
- MEDS - Metoprolol, Lisinopril, Valium
- SHx- Never smoker, retired event planner
- PE
  - Negative
- Urinalysis
  - Straw color
  - Dipstick: - nitrite, - leukocyte esterase and trace blood
  - Microscopic - 7 RBC/hpf , 0 WBC/hpf or bacteria
- Urine culture
  - Negative

- Mr. TW is a 71 year-old white male found to have microscopic hematuria on 2 urinalyses after recent hospitalization for exacerbation of congestive heart failure (CHF). No pain or symptoms noted.
- PMH - CHF, HTN, panic attacks
- MEDS - Metoprolol, Lisinopril, valium
- SHx- Never smoker, retired event planner
- PE
  - Negative
- Urinalysis
  - Straw color
  - Dipstick: - nitrite, - leukocyte esterase and trace blood
  - Microscopic - 7 RBC/hpf , 0 WBC/hpf or bacteria
- Urine culture
  - Negative

- **What urine-based test would you order next and why?**

❑ Urinary Cytology

❑ Immunocyt/uCyt+

❑ Urovysion (FISH)

❑ BTAstat

❑ Bladdercheck (NMP22)

❑ Other;____________________

❑ None

- Mr. TW is a 71 year-old white male found to have microscopic hematuria on 2 urinalyses after recent hospitalization for exacerbation of congestive heart failure (CHF). No pain or symptoms noted.
- PMH - CHF, HTN, panic attacks
- MEDS - Metoprolol, Lisinopril, valium
- SHx- Never smoker, retired event planner
- PE
  - Negative
- Urinalysis
  - Straw color
  - Dipstick: - nitrite, - leukocyte esterase and trace blood
  - Microscopic - 7 RBC/hpf , 0 WBC/hpf or bacteria
- Urine culture
  - Negative
- Voided urinary cytology - atypia

- **What additional urine-based test would you order next and why?**

- ❑ Immunocyt/uCyt+
- ❑ Urovysion (FISH)
- ❑ BTAstat
- ❑ Bladdercheck (NMP22)
- ❑ Other;____________________
- ❑ None

- Mr. TW is a 71 year-old white male found to have microscopic hematuria on 2 urinalyses after recent hospitalization for exacerbation of congestive heart failure (CHF). No pain or symptoms noted.
- PMH - CHF, HTN, panic attacks
- MEDS - Metoprolol, Lisinopril, valium
- SHx- Never smoker, retired event planner
- PE
  - Negative
- Urinalysis
  - Straw color
  - Dipstick: - nitrite, - leukocyte esterase and trace blood
  - Microscopic - 7 RBC/hpf , 0 WBC/hpf or bacteria
- Urine culture
  - Negative
- Voided urinary cytology - atypia

- **What diagnostic tests would you perform next?**

❑Office cystoscopy

❑CT scan of abdomen/pelvis

❑Transurethral resection of bladder tumor (TURBT)

❑Other;____________________

❑None

- Mr. TW is a 71 year-old white male found to have microscopic hematuria on 2 urinalyses after recent hospitalization for exacerbation of congestive heart failure (CHF). No pain or symptoms noted.
- PMH - CHF, HTN, panic attacks
- MEDS - Metoprolol, Lisinopril, valium
- SHx- Never smoker, retired event planner
- PE
  - Negative
- Urinalysis
  - Straw color
  - Dipstick: - nitrite, - leukocyte esterase and trace blood
  - Microscopic - 7 RBC/hpf , 0 WBC/hpf or bacteria
- Urine culture
  - Neg
- Voided urinary cytology
  - Atypia
- Oncuria risk score 47% (positive)

Now, noting the Oncuria risk score, would this change the urine-based test you ordered?

❑Yes ❑No

If yes, then what would you order and why?

❑Urinary Cytology

❑Immunocyt/uCyt+

❑Urovysion (FISH)

❑BTAstat

❑Bladdercheck (NMP22)

❑Other; ____________________________

❑None

- Mr. TW is a 71 year-old white male found to have microscopic hematuria on 2 urinalyses after recent hospitalization for exacerbation of congestive heart failure (CHF). No pain or symptoms noted.
- PMH - CHF, HTN, panic attacks
- MEDS - Metoprolol, Lisinopril, valium
- SHx- Never smoker, retired event planner
- PE
  - Negative
- Urinalysis
  - Straw color
  - Dipstick: - nitrite, - leukocyte esterase and trace blood
  - Microscopic - 7 RBC/hpf , 0 WBC/hpf or bacteria
- Urine culture
  - Neg
- Voided urinary cytology
  - Atypia
- Oncuria risk score 47% (positive)

- **Now, noting Oncuria risk score, what diagnostic tests would you perform next?**

❑ Office cystoscopy

❑ CT scan of abdomen/pelvis

❑ Transurethral resection of bladder tumor (TURBT)

❑ Other; ______________________

❑ None

FINAL RESULT:
Cytology atypia and bladderchek (NMP22) negative
Office cystoscopy noted a bladder mass
CT scan noted bladder mass and negative upper tract
TURBT demonstrated a 3 cm mass - pathology T1 HG TCC (UC)

If clinically launched and is as sensitive as described in the manuscripts, how could Oncuria change the way you use urine-based bladder cancer detection assays?
Did or could Oncuria change the way you practice based on this case?

- Mrs. DS is a 62 year-old Asian female found to have microscopic hematuria by PCP on two urinalyses. No pain or symptoms noted.
- PMH - None
- MEDS - None
- SHx- 63 pack years and from a household with smokers, exposed to asbestos dental chemicals as a dental hygienist
- PE
  - Negative
- Urinalysis
  - Straw color
  - Dipstick: - nitrite, - leukocyte esterase and - blood
  - Microscopic - 3 RBC/hpf, 0 WBC/hpf or bacteria
- Urine culture
  - <10,000 normal flora

- Mrs. DS is a 62 year-old Asian female found to have microscopic hematuria by PCP on two urinalyses. No pain or symptoms noted.
- PMH - None
- MEDS - None
- SHx- 63 pack years and from the age of 13-44 years, from a household with smokers, exposed to asbestos dental chemicals as a dental hygienist
- PE
  - Negative
- Urinalysis
  - Straw color
  - Dipstick: - nitrite, - leukocyte esterase and - blood
  - Microscopic - 3 RBC/hpf, 0 WBC/hpf or bacteria
- Urine culture
  - <10,000 normal flora

- **What urine-based test would you order next and why?**

❑ Urinary Cytology

❑ Immunocyt/uCyt+

❑ Urovysion (FISH)

❑ BTAstat

❑ Bladdercheck (NMP22)

❑ Other; ____________________

❑ None

- Mrs. DS is a 62 year-old Asian female found to have microscopic hematuria by PCP on two urinalyses. No pain or symptoms noted.
- PMH - None
- MEDS - None
- SHx- 63 pack years and from the age of 13-44 years, from a household with smokers, exposed to asbestos dental chemicals as a dental hygienist
- PE
  - Negative
- Urinalysis
  - Straw color
  - Dipstick: - nitrite, - leukocyte esterase and - blood
  - Microscopic - 3 RBC/hpf, 0 WBC/hpf or bacteria
- Urine culture
  - <10,000 normal flora
- Voided urinary cytology - negative

- **What additional urine-based test would you order next and why?**

- [ ] Immunocyt/uCyt+
- [ ] Urovysion (FISH)
- [ ] BTAstat
- [ ] Bladdercheck (NMP22)
- [ ] Other; ______________________
- [ ] None

- Mrs. DS is a 62 year-old Asian female found to have microscopic hematuria by PCP on two urinalyses. No pain or symptoms noted.
- PMH - None
- MEDS - None
- SHx- 63 pack years and from the age of 13-44 years, from a household with smokers, exposed to asbestos dental chemicals as a dental hygienist
- PE
  - Negative
- Urinalysis
  - Straw color
  - Dipstick: - nitrite, - leukocyte esterase and - blood
  - Microscopic - 3 RBC/hpf, 0 WBC/hpf or bacteria
- Urine culture
  - <10,000 normal flora
- Voided urinary cytology - negative

- **What diagnostic tests would you perform next?**

❑Office cystoscopy

❑CT scan of abdomen/pelvis

❑Transurethral resection of bladder tumor (TURBT)

❑Other; ______________________

❑None

- Mrs. DS is a 62 year-old Asian female found to have microscopic hematuria by PCP on two urinalyses. No pain or symptoms noted.
- PMH - None
- MEDS - None
- SHx- 63 pack years and from the age of 13-44 years, from a household with smokers, exposed to asbestos dental chemicals as a dental hygienist
- PE
  - Negative
- Urinalysis
  - Straw color
  - Dipstick: - nitrite, - leukocyte esterase and - blood
  - Microscopic - 3 RBC/hpf, 0 WBC/hpf or bacteria
- Urine culture
  - <10,000 normal flora
- Voided urinary cytology - negative
- Oncuria risk score 2% (negative)

Now, noting the Oncuria risk score, would this change the urine-based test you ordered?

❑Yes ❑No

If yes, then what would you order and why?

❑Urinary Cytology

❑Immunocyt/uCyt+

❑Urovysion (FISH)

❑BTAstat

❑Bladdercheck (NMP22)

❑Other; ______________________

❑None

- Mrs. DS is a 62 year-old Asian female found to have microscopic hematuria by PCP on two urinalyses. No pain or symptoms noted.
- PMH - None
- MEDS - None
- SHx- 63 pack years and from a household with smokers, exposed to asbestos dental chemicals as a dental hygienist
- PE
  - Negative
- Urinalysis
  - Straw color
  - Dipstick: - nitrite, - leukocyte esterase and - blood
  - Microscopic - 3 RBC/hpf, 0 WBC/hpf or bacteria
- Urine culture
  - <10,000 normal flora
- Voided urinary cytology - negative
- Oncuria risk score 2% (negative)

- **Noting Oncuria risk score, what diagnostic tests would you perform next?**

❑ Office cystoscopy

❑ CT scan of abdomen/pelvis

❑ Transurethral resection of bladder tumor (TURBT)

❑ Other; ____________________

❑ None

FINAL RESULT:
Cytology and bladderchek (NMP22) negative
Office cystoscopy negative
CT scan negative

If clinically launched and is as sensitive as described in the manuscripts, how could Oncuria change the way you use urine-based bladder cancer detection assays?
Did or could Oncuria change the way you practice based on this case?

Bladder Cancer Surveillance

- Mr. DE is an 81 year-old white man with a history of bladder cancer (cT3NxMx HG) diagnosed in 2015. Treated with tri-modal therapy ~ 9 months ago and is on every 3 month cystoscopy for tumor surveillance. No symptoms noted.
- PMH - HTN, Arthritis, Hypercholesterolemia
- MEDS - HCTZ, Pravachol, Tylenol
- SHx- Former smoker (40 pack years)
- PE
  - Negative
- Urinalysis
  - Pale yellow
  - Dipstick - Negative
  - Microscopic - Negative

- Mr. DE is an 81 year-old white man with a history of bladder cancer (cT3NxMx HG) diagnosed in 2015. Treated with tri-modal therapy ~ 9 months ago and is on every 3 month cystoscopy for tumor surveillance. No symptoms noted.
- PMH - HTN, Arthritis, Hypercholesterolemia
- MEDS - HCTZ, Pravachol, Tylenol
- SHx- Former smoker (40 pack years)
- PE
  - Negative
- Urinalysis
  - Pale yellow
  - Dipstick - Negative
  - Microscopic - Negative

- **What urine-based test would you order next and why?**

❑ Urinary cytology

❑ Immunocyt/uCyt+

❑ Urovysion (FISH)

❑ BTAstat

❑ Bladdercheck (NMP22)

❑ Other; ____________________

❑ None

- Mr. DE is a 81 year-old white male with a history of bladder cancer (cT3NxMx HG) diagnosed in 2015. Treated with tri-modal therapy ~ 9 months ago and is on every 3 month cystoscopy for tumor surveillance. No symptoms noted.
- PMH - HTN, Arthritis, Hypercholesterolemia
- MEDS - HCTZ, Pravachol, Tylenol
- SHx- Former smoker (40 pack years)
- PE
  - Negative
- Urinalysis
  - Pale yellow
  - Dipstick - Negative
  - Microscopic - Negative
- Voided urinary cytology - negative

- **What additional urine-based test would you order next and why?**

- ❑ Immunocyt/uCyt+
- ❑ Urovysion (FISH)
- ❑ BTAstat
- ❑ Bladdercheck (NMP22)
- ❑ Other; ________________
- ❑ None

- Mr. DE is a 81 year-old white male with a history of bladder cancer (cT3NxMx HG) diagnosed in 2015. Treated with tri-modal therapy ~ 9 months ago and is on every 3 month cystoscopy for tumor surveillance. No symptoms noted.
- PMH - HTN, Arthritis, Hypercholesterolemia
- MEDS - HCTZ, Pravachol, Tylenol
- SHx- Former smoker (40 pack years)
- PE
  - Negative
- Urinalysis
  - Pale yellow
  - Dipstick - Negative
  - Microscopic - Negative
- Voided urinary cytology - negative

- **What diagnostic tests would you perform next?**

❑ Office cystoscopy

❑ CT scan of abdomen/pelvis

❑ Transurethral resection of bladder tumor (TURBT)

❑ Other; ________________

❑ None

- Mr. DE is an 81 year-old white male with a history of bladder cancer (cT3NxMx HG) diagnosed in 2015. Treated with tri-modal therapy ~ 9 months ago and is on every 3 month cystoscopy for tumor surveillance. No symptoms noted.
- PMH - HTN, Arthritis, Hypercholesterolemia
- MEDS - HCTZ, Pravachol, Tylenol
- SHx- Former smoker (40 pack years)
- PE
  - Negative
- Urinalysis
  - Pale yellow
  - Dipstick - Negative
  - Microscopic - Negative
- Oncuria risk score 45% (negative)

Now, noting the Oncuria risk score, would this change the urine-based test you ordered?

❑Yes ❑No

If yes, then what would you order and why?

❑Urinary Cytology

❑Immunocyt/uCyt+

❑Urovysion (FISH)

❑BTAstat

❑Bladdercheck (NMP22)

❑Other; ______________________

❑None

- Mr. DE is an 81 year-old white male with a history of bladder cancer (cT3NxMx HG) diagnosed in 2015. Treated with tri-modal therapy ~ 9 months ago and is on every 3 month cystoscopy for tumor surveillance. No symptoms noted.
- PMH - HTN, Arthritis, Hypercholesterolemia
- MEDS - HCTZ, Pravachol, Tylenol
- SHx- Former smoker (40 pack years)
- PE
  - Negative
- Urinalysis
  - Pale yellow
  - Dipstick - Negative
  - Microscopic - Negative
- Oncuria risk score 45% (negative)

- **Now, noting the Oncuria risk score, what diagnostic tests would you perform next?**

❑ Office cystoscopy

❑ CT scan of abdomen/pelvis

❑ Transurethral resection of bladder tumor (TURBT)

❑ Other; ________________

❑ None

FINAL RESULT:
Cytology and bladderchek (NMP22) negative
Office cystoscopy negative
CT scan not performed

Note - 6 months later the patient had a recurrence of Ta LG TCC (UC) and Oncuria risk score at that time was 49% (positive)

If clinically launched and is as sensitive as described in the manuscripts, how could Oncuria change the way you use urine-based bladder cancer detection assays?
Did or could Oncuria change the way you practice based on this case?

- Mr. ST is a 65 year-old white male with a history of bladder cancer (cT1NxMx HG) diagnosed in 2011. Treated with TURBT and adjuvant intravesical BCG. Presents for month 12 office cystoscopy for tumor surveillance. No symptoms.
- PMH - DM
- MEDS - Glyburide
- SHx- Former smoker (unknown pack years)
- PE
  - Negative
- Urinalysis
  - Pale yellow
  - Dipstick - Negative
  - Microscopic - 5 RBC/hpf

- Mr. ST is a 65 year-old white male with a history of bladder cancer (cT1NxMx HG) diagnosed in 2011. Treated with TURBT and adjuvant intravesical BCG. Presents for month 12 office cystoscopy for tumor surveillance. No symptoms.
- PMH - DM
- MEDS - Glyburide
- SHx- Former smoker (unknown pack years)
- PE
  - Negative
- Urinalysis
  - Pale yellow
  - Dipstick - Negative
  - Microscopic - 5 RBC/hpf

- **What urine-based test would you order next and why?**

- [ ] Urinary cytology
- [ ] Immunocyt/uCyt+
- [ ] Urovysion (FISH)
- [ ] BTAstat
- [ ] Bladdercheck (NMP22)
- [ ] Other; ____________________
- [ ] None

- Mr. ST is a 65 year-old white male with a history of bladder cancer (cT1NxMx HG) diagnosed in 2011. Treated with TURBT and adjuvant intravesical BCG. Presents for month 12 office cystoscopy for tumor surveillance. No symptoms.
- PMH - DM
- MEDS - Glyburide
- SHx- Former smoker (unknown pack years)
- PE
  - Negative
- Urinalysis
  - Pale yellow
  - Dipstick - Negative
  - Microscopic - 5 RBC/hpf
- Voided urinary cytology - positive

- **What additional urine-based test would you order next and why?**

- ☐ Immunocyt/uCyt+
- ☐ Urovysion (FISH)
- ☐ BTAstat
- ☐ Bladdercheck (NMP22)
- ☐ Other; ________________
- ☐ None

- Mr. ST is a 65 year-old white male with a history of bladder cancer (cT1NxMx HG) diagnosed in 2011. Treated with TURBT and adjuvant intravesical BCG. Presents for month 12 office cystoscopy for tumor surveillance. No symptoms.
- PMH - DM
- MEDS - Glyburide
- SHx- Former smoker (unknown pack years)
- PE
  - Negative
- Urinalysis
  - Pale yellow
  - Dipstick - Negative
  - Microscopic - 5 RBC/hpf
- Voided urinary cytology - positive

- **What diagnostic tests would you perform next?**

❑ Office cystoscopy

❑ CT scan of abdomen/pelvis

❑ Transurethral resection of bladder tumor (TURBT)

❑ Other; ________________

❑ None

- Mr. ST is a 65 year-old white male with a history of bladder cancer (cT1NxMx HG) diagnosed in 2011. Treated with TURBT and adjuvant intravesical BCG. Presents for month 12 office cystoscopy for tumor surveillance. No symptoms.
- PMH - DM
- MEDS - Glyburide
- SHx- Former smoker (unknown pack years)
- PE
  - Negative
- Urinalysis
  - Pale yellow
  - Dipstick - Negative
  - Microscopic - 5 RBC/hpf
- Oncuria risk score 99% (positive)

Now, noting the Oncuria risk score, would this change the urine-based test you ordered?

❑Yes ❑No

If yes, then what would you order and why?

❑Urinary Cytology

❑Immunocyt/uCyt+

❑Urovysion (FISH)

❑BTAstat

❑Bladdercheck (NMP22)

❑Other; ____________________

❑None

- Mr. ST is a 65 year-old white male with a history of bladder cancer (cT1NxMx HG) diagnosed in 2011. Treated with TURBT and adjuvant intravesical BCG. Presents for month 12 office cystoscopy for tumor surveillance. No symptoms.
- PMH - DM
- MEDS - Glyburide
- SHx- Former smoker (unknown pack years)
- PE
  - Negative
- Urinalysis
  - Pale yellow
  - Dipstick - Negative
  - Microscopic - 5 RBC/hpf
- Oncuria risk score 99% (positive)

- **Now, noting the Oncuria risk score, what diagnostic tests would you perform next?**

- [ ] Office cystoscopy
- [ ] CT scan of abdomen/pelvis
- [ ] Transurethral resection of bladder tumor (TURBT)
- [ ] Other;____________________
- [ ] None

FINAL RESULT:
Cytology positive and bladderchek (NMP22) negative
Office cystoscopy noted a bladder mass
CT scan noted bladder mass and negative upper tract
TURBT demonstrated a recurrent 1 cm mass - pathology T2 HG TCC (UC)

If clinically launched and is as sensitive as described in the manuscripts, how could Oncuria change the way you use urine-based bladder cancer detection assays?
Did or could Oncuria change the way you practice based on this case?

- Mrs. EM is a 76 year-old white female with a history of bladder cancer (CisNxMx HG) diagnosed in 2017. Treated with TURBT and clinical trial with intravesical therapy. Presents at month 7 with gross hematuria. Missed month 6 office cystoscopy. Otherwise no symptoms.
- PMH - None
- MEDS - None
- SHx- Former smoker (unknown pack years)
- PE
  - Negative
- Urinalysis
  - Pink
  - Dipstick - +blood, - nitrite, - leukocyte esterase
  - Microscopic - TNTC RBC/hpf, 5-10 WBC/hpf, +1 bacteria

- Mrs. EM is a 76 year-old white female with a history of bladder cancer (CisNxMx HG) diagnosed in 2017. Treated with TURBT and clinical trial with intravesical therapy. Presents at month 7 with gross hematuria. Missed month 6 office cystoscopy. Otherwise no symptoms.
- PMH - None
- MEDS - None
- SHx- Former smoker (unknown pack years)
- PE
  - Negative
- Urinalysis
  - Pink
  - Dipstick - +blood, - nitrite, - leukocyte esterase
  - Microscopic - TNTC RBC/hpf, 5-10 WBC/hpf, +1 bacteria

- **What urine-based test would you order next and why?**

❑Urinary cytology

❑Immunocyt/uCyt+

❑Urovysion (FISH)

❑BTAstat

❑Bladdercheck (NMP22)

❑Other; ______________________

❑None

- Mrs. EM is a 76 year-old white female with a history of bladder cancer (CisNxMx HG) diagnosed in 2017. Treated with TURBT and clinical trial with intravesical therapy. Presents at month 7 with gross hematuria. Missed month 6 office cystoscopy. Otherwise no symptoms.
- PMH - None
- MEDS - None
- SHx- Former smoker (unknown pack years)
- PE
  - Negative
- Urinalysis
  - Pink
  - Dipstick - +blood, - nitrite, - leukocyte esterase
  - Microscopic - TNTC RBC/hpf, 5-10 WBC/hpf, +1 bacteria
- Voided urinary cytology - positive

- **What additional urine-based test would you order next and why?**

- ❑ Immunocyt/uCyt+
- ❑ Urovysion (FISH)
- ❑ BTAstat
- ❑ Bladdercheck (NMP22)
- ❑ Other; ____________________
- ❑ None

- Mrs. EM is a 76 year-old white female with a history of bladder cancer (CisNxMx HG) diagnosed in 2017. Treated with TURBT and clinical trial with intravesical therapy. Presents at month 7 with gross hematuria. Missed month 6 office cystoscopy. Otherwise no symptoms.
- PMH - None
- MEDS - None
- SHx- Former smoker (unknown pack years)
- PE
  - Negative
- Urinalysis
  - Pink
  - Dipstick - +blood, - nitrite, - leukocyte esterase
  - Microscopic - TNTC RBC/hpf, 5-10 WBC/hpf, +1 bacteria
- Voided urinary cytology - positive

- **What diagnostic tests would you perform next?**

❑Office cystoscopy

❑CT scan of abdomen/pelvis

❑Transurethral resection of bladder tumor (TURBT)

❑Other; ____________________

❑None

- Mrs. EM is a 76 year-old white female with a history of bladder cancer (CisNxMx HG) diagnosed in 2017. Treated with TURBT and clinical trial with intravesical therapy. Presents at month 7 with gross hematuria. Missed month 6 office cystoscopy. Otherwise no symptoms.
- PMH - None
- MEDS - None
- SHx- Former smoker (unknown pack years)
- PE
  - Negative
- Urinalysis
  - Pink
  - Dipstick - +blood, - nitrite, - leukocyte esterase
  - Microscopic - TNTC RBC/hpf, 5-10 WBC/hpf, +1 bacteria
- Oncuria risk score 71% (positive)

Now, noting the Oncuria risk score, would this change the urine-based test you ordered?

❑Yes ❑No

If yes, then what would you order and why?

❑Urinary Cytology

❑Immunocyt/uCyt+

❑Urovysion (FISH)

❑BTAstat

❑Bladdercheck (NMP22)

❑Other; ___________________________

❑None

- Mrs. EM is a 76 year-old white female with a history of bladder cancer (CisNxMx HG) diagnosed in 2017. Treated with TURBT and clinical trial with intravesical therapy. Presents at month 7 with gross hematuria. Missed month 6 office cystoscopy. Otherwise no symptoms.
- PMH - None
- MEDS - None
- SHx- Former smoker (unknown pack years)
- PE
  - Negative
- Urinalysis
  - Pink
  - Dipstick - +blood, - nitrite, - leukocyte esterase
  - Microscopic - TNTC RBC/hpf, 5-10 WBC/hpf, +1 bacteria
- Oncuria risk score 71% (positive)

- **Now, noting the Oncuria risk score, what diagnostic tests would you perform next?**

❑ Office cystoscopy

❑ CT scan of abdomen/pelvis

❑ Transurethral resection of bladder tumor (TURBT)

❑ Other;____________________

❑ None

FINAL RESULT:
Cytology and bladderchek (NMP22) positive
Office cystoscopy noted a bladder mass
CT scan noted bladder mass and negative upper tract
TURBT demonstrated a recurrent 1 cm mass - pathology T1 HG TCC (UC)

If clinically launched and is as sensitive as described in the manuscripts, how could Oncuria change the way you use urine-based bladder cancer detection assays?
Did or could Oncuria change the way you practice based on this case?

Final thoughts on urine-based bladder cancer biomarkers?
Final thoughts on Oncuria?

Thank you for your time
