# Supplementary material for: Clinical Utility of Oncuria™, a Multiplexed Liquid Biopsy for the Non-Invasive Detection of Bladder Cancer—A Pilot Study
Source: Diagnostics (Basel). 2022 Jan 6;12(1):131. doi: 10.3390/diagnostics12010131 (PMC8774623; doi:10.3390/diagnostics12010131)
Supplement: Supplementary file 1 [file diagnostics-12-00131-s001.zip › Supplementary File S2.pdf]

Demographics and Practice Characteristics of Fifteen Urologists Responding to Urine-Based Bladder Cancer Detection Assay Screening Attitude and Behavior Survey

**Variable**	**Characteristics**	**Frequency (%)**	**National Trends (%) [12]**
Age (years)			
	30-45	46	28
	46-60	26	42.4
	$\geq$65	28	29.6
Gender			
	Male	86	90.1
	Female	14	9.9
Race			
	White	73	84.7
	Non-white	27	15.3
Practice specialty			
	General Urology	80	65
	Urologic Oncology	20	12.4
Practice type			
	Group/Academic	40	27.3
	Group/Private	33	43.9
	Hospital based	20	28.8
	Solo practice	7	9.4
Office setting			
	Urban/Metropolitan	86	89.6
	Rural/Non-Metropolitan	14	10.4
% Medicare in practice			
	<25%	13	---
	26-50	46	---
	>50%	71	---
% Medicaid in practice			
	<25%	86	---
	26-50	14	---
	>50%	0	---
